# Development and evaluation of e-mental health interventions to reduce stigmatization of suicidality – a study protocol

**DOI:** 10.1186/s12888-019-2137-0

**Published:** 2019-05-17

**Authors:** Mareike Dreier, Julia Ludwig, Martin Härter, Olaf von dem Knesebeck, Johanna Baumgardt, Thomas Bock, Jörg Dirmaier, Alison J. Kennedy, Susan A. Brumby, Sarah Liebherz

**Affiliations:** 1 Department of Medical Psychology, Center for Psychosocial Medicine, University Medical Center Hamburg-Eppendorf, Martinistr. 52, Building W26, 20246 Hamburg, Germany; 2 Department of Medical Sociology, Center for Psychosocial Medicine, University Medical Center Hamburg-Eppendorf, Hamburg, Germany; 3 Department of Psychiatry and Psychotherapy, Center for Psychosocial Medicine, University Medical Center Hamburg-Eppendorf, Hamburg, Germany; 4 National Centre for Farmer Health, School of Medicine, Deakin Unversity, Waurn Ponds, Victoria Australia; 5 Western District Health Service, Hamilton, Victoria Australia

**Keywords:** Suicide, Stigma, Mental health literacy, E-mental health, Telephone survey, Mixed methods research

## Abstract

**Background:**

Worldwide, approximately 800,000 persons die by suicide every year; with rates of suicide attempts estimated to be much higher. Suicidal persons often suffer from a mental disorder but stigma, lack of available and suitable support, and insufficient information on mental health limit help seeking. The use of internet-based applications can help individuals inform themselves about mental disorders, assess the extent of their own concerns, find local treatment options, and prepare for contact with health care professionals. This project aims to develop and evaluate e-mental health interventions to improve knowledge about suicidality and to reduce stigmatization of those affected. In developing these interventions, a representative telephone survey was conducted to detect knowledge gaps and stigmatizing attitudes in the general population.

**Methods:**

First, a national representative telephone survey with *N* = 2000 participants in Germany was conducted. Second, e-mental health interventions are developed to address knowledge gaps and public stigma detected in the survey. These comprise an evidence-based health information package about suicidality, information on regional support services, a self-administered depression test—including suicidality—and an interactive online intervention including personal stories. The development is based on a trialogical exchange of experience between persons affected by suicidality, relatives of affected persons, and clinical experts. Australian researchers who developed an e-mental health intervention for individuals affected by rural suicide were invited to a workshop in order to contribute their knowledge and expertise. Third, the online intervention will be evaluated by a mixed methods design.

**Discussion:**

From representative telephone survey data, content can be developed to address specific attitudes and knowledge via the e-mental health interventions. These interventions will be easily accessed and provide an opportunity to reach people who tend not to seek professional services, prefer to inform themselves in advance and/or wish to remain anonymous. Evaluation of the online intervention will provide information on any changes in participants’ self-stigma and perceived-stigma of suicidality, and any increase in participants’ knowledge on suicidality or self-efficacy expectations.

**Trial registration:**

German Clinical Trial Register DRKS00015071 on August 6, 2018.

## Background

Worldwide, more than 800,000 people die by suicide every year [1]. Compared to other regions in the world, Europe had the highest suicide rate in 2016 (15.4 per 100,000 population). In Germany, approximately 10,000 people die by suicide every year (13.6 per 100,000 population in 2016) [2]. Rates of suicide attempts are estimated to be much higher: For each person who dies by suicide, it is estimated that more than 20 others attempt suicide [1]. Since suicide is a sensitive issue, it is difficult to quantify exact numbers of suicide attempts and suicide deaths. The World Health Organization [1] assumes that suicide is under-reported. Even in countries with good reporting systems suicide may be missclassified as another cause of death [1]. Around 90% of people dying by suicide in Western industrialised countries have been diagnosed with a mental health condition, particularly affective disorders, substance-related disorders, schizophrenia, and personality disorders [3–5].

Suicide is a complex issue with multiple contributing factors. Although many people who die by suicide experience a diagnosed mental health condition, suicidality is also influenced by situational factors, e.g. physical illness or injury, financial problems, or other life crises [1]. Effective treatment of poor mental health is impeded by stigma [6–8], lack of available and suitable support and insufficient information on mental health [9].

In terms of stigma, public, self, and perceived stigma can be distinguished. This project targets all three dimensions of stigma. Public stigma comprises stigmatizing reactions and attitudes of the general public towards members of a particular social group (for example persons with suicidal thoughts). Negative beliefs about this group (“Persons with suicidal thoughts have a weak will”) and negative emotional reactions (“I feel annoyed by that”) can lead to discriminating behaviour e.g. withholding help [10, 11]. Self-stigma implies that negative emotional reactions, or stereotypes are internalized which means affected persons apply them to themselves (“Because I had thoughts of ending my life, I have a weak will.”), which leads to lower self-esteem and self-efficacy [12]. While public stigma can be seen as a direct social jugdement, perceived stigma is the expected negative reaction of the public by an individual in response to their mental health condition. This can effect self-concept, functioning, and adequate health care utilization [7, 13, 14].

In Germany, less than half of all people experiencing a mental health condition report having used any provider or service for mental health reasons [15]. Easy access opportunities to inform individuals about their health and to build personal capacity to manage their health could help people who do not have, or want access to traditional health services.

There is evidence that a range of interventions (e.g. interventions in educational settings, or via information websites) can improve knowledge about mental disorders, and support recognition, management and prevention efforts [16]. Mental health literacy has focused on the recognition of mental illnesses, knowledge about risk factors and causes, about self-help and professional help or knowledge on prevention of mental disorders with the aim of enabling help-seeking [17, 18]. Thus, improving mental health literacy is part of the e-mental health interventions.

Due to the widespread use of modern communication technologies (in 2017, 81% of the German population were internet users [19]), new possibilities arise for improving support for people with mental health problems or other health crises. For example, the US National Institute of Mental Health has recommended the development of innovative treatment approaches that are both affordable and accessible to a large population [20]. Modern communication technologies provide this opportunity.

Recent studies report that the use of new media (e.g. the internet) can be effective in both treating and preventing mental disorders [21–25]. Internet-based applications can help people inform themselves about mental disorders, to assess the extent of their own concerns, to find local treatment options, and to prepare for contact with health care professionals. Self-help programs can significantly contribute to improve symptoms [26]. As demonstrated by the results of the OSPI-Europe suicide prevention program [27], awareness campaigns can help to reduce stigma and foster openness towards seeking and accepting professional help. A recent review on suicide prevention strategies [28] shows evidence for the effectiveness of restricting access to the means of suicide (e.g. firearms, analgesic medication), education campaigns in schools, specific psychopharmacological and psychotherapeutic approaches, and the aftercare of persons with a previous suicide attempt.

There has been limited evaluation of the effectiveness of e-mental health approaches to suicide prevention [28]. In a randomized controlled trial, unguided online self-help interventions aiming to reduce suicidal ideation showed a reduction in suicidal thoughts compared to a waitlist control group in a Dutch sample [29]. In a more recent Australian randomized controlled trial (online self-help intervention based on the Dutch program vs. attention-matched control program) no group differences in suicidal thinking were found [30].

For online interventions aiming to reduce suicide stigma, a recent study has been undertaken in Australia [31]. However, the study’s outcomes are not yet available ([32], Kennedy AJ, Brumby SA, Versace VL, Brumby-Rendell T: The ripple effect: a digital intervention to reduce suicide stigma among farming men, submitted). It is presumed that stigma is associated with higher prevalence of suicide [33]. There is some evidence for a link between self-stigma and suicidality. A recent longitudinal study shows that self-stigma impedes the lives of persons with mental disorders by increasing suicidality. Suicide prevention could be improved by interventions that reduce stigma [34, 35].

## Methods

### Study aims

This project aims to develop and evaluate e-mental health interventions in order to improve knowledge and to reduce suicide stigma. The target group are persons with suicidal thoughts or suicide attempts in the past, their relatives, and persons generally interested in the topic.

The interventions will be integrated in the evidence-based German e-mental health portal https://www.psychenet.de/ [36], established since 2011, and currently supported by the German Association for Psychiatry, Psychotherapy and Psychosomatics (DGPPN). The portal provides evidence-based health information on several mental illnesses and general topics concerning mental health, decision aids and self-tests on mental disorders (e.g. depression, somatoform disorders, eating and anxiety disorders), as well as information on the German health care system, and an awareness campaign on mental health. People affected by mental disorders and their relatives were involved in the development process. *Psychenet.de* intends to increase mental health literacy to empower users in managing mental health challenges [37, 38].

The project is focused on the following aims:

1. *To realize a representative population survey of knowledge and attitudes towards suicidality in Germany:* Knowledge about causes, signs, support, and treatment options of suicidality as well as attitudes towards suicidal persons (stigma) will be evaluated in order to deduce knowledge gaps and stigmatizing attitudes which can be addressed by the e-mental health interventions.
2. *To develop e-mental health interventions:* Two e-mental health interventions will be developed: (a) an extension of the existing e-mental health portal psychenet.de focused on suicidality, and (b) an interactive online intervention focused on reducing suicide stigma, which will be available on a subdomain of *psychenet.de*. Evidence-based health information about suicidality and information on regional support services for severe mental or suicidal crises will be developed for both. One item assessing suicidal thoughts will be added to the existing self-test for depressive disorders (PHQ-9 [39]) and will be uploaded on *psychenet.de*. The interactive online intervention, inspired by the Australian project *The Ripple Effect* [31, 40], will consist of reports by persons with an experience of suicide in the form of 10–20 videos (duration: 2–5 min) and written experience reports. Psychoeducative elements and strategies to deal with suicide stigma will be developed for different target groups.
3. *To evaluate the e-mental health interventions:* For evaluating the extension of the existing e-mental health portal *psychenet.de* (a) we will use web analytics. For evaluating the online intervention (b), participants will be recruited via *psychenet.de*. In a pre-post survey, we will evaluate to what extent an interactive online intervention reduces self-stigma and perceived-stigma, improves suicide literacy, self-efficacy expectations, and affects the participants’ intention to seek help (outcome evaluation). The participants’ evaluation of the content of the online intervention (e.g. satisfaction, helpful aspects) will also be assessed immediately after completing the intervention, as well as in a follow-up survey 12–26 weeks after completing the intervention (process evaluation). While the pre-post survey will primarily collect quantitative data, the follow-up survey will provide qualitative data by semi-structured telephone interviews.

## Aim 1: representative population survey

### Study design

In April and May 2018, a cross-sectional telephone survey with *N* = 2000 persons was conducted in Germany. The survey dealt with attitudes and knowledge towards suicidality and was conducted by a professional market- and social-research institute. Time taken to do the survey was approximately 20 min.

A case vignette with signs and symptoms of a person with suicidal thoughts was presented to the participants. The vignettes systematically varied in gender (female vs. male), age (younger vs. older person) and disorder (mental disorder: depression, somatic disorder: cancer) resulting in eight different vignettes and approximately 250 respondents for each combination. The vignettes were developed by the project team and discussed with physicians, psycho-oncologists, psychotherapists, and people with lived experience. They were audio-recorded with a trained speaker to increase reliability and to counteract possible interviewer effects. The vignettes are:

#### Mental disorder (depression)

The 32−/73-year old Johanna D./Johannes D. has been feeling depressed and sad for a couple of months. Ms./Mr. D. feels useless, has the impression of doing everything wrong and has lost any interest in the things that usually brought joy to her/him. She/he doubts that her/his life has any meaning and, with increasing frequency, thinks about taking her/his own life.

#### Somatic disorder (cancer)

The 32−/73-year old Johanna D./Johannes D. has been told a couple of months ago, that she/he is suffering from cancer. Currently Ms./Mr. D. is constantly exhausted and suffers from nausea and pain. She/He is feeling hopeless and fears a progression of the disease. She/he doubts that her/his life has any meaning and, with increasing frequency, thinks about taking her/his own life.

### Study sample: inclusion and exclusion criteria

The sample consisted of adults aged 18 and older, living in private households with a landline or cell phone in Germany. In order to reach all groups of persons, telephone numbers were drawn from all registered telephone numbers at random. Ex-directory households and cell phone numbers were included via computer-generated numbers. Persons younger than 18 years or those with neither a mobile phone number nor a landline number were excluded. As this is a questionnaire in German, people who did not understand German were also excluded.

### Data collection

The population telephone survey was conducted by the market- and social-research institute USUMA which is located in Berlin. Data was collected with the aid of a computer assisted telephone interview (CATI). To get representative data for the adult residential population in Germany, the sample consisted of randomly generated mobile phone numbers and non-registered numbers as well as randomly selected registered telephone numbers. In households with more than one resident, the Kish selection grid was used to randomly select the target person [41]. This multilevel sample design ensured that in every household with multiple residents, each person had the same chance to be selected for the survey. Collected data was transferred to the University Medical Center Hamburg-Eppendorf for data analyses.

### Measures

The questionnaire asked about attitudes and knowledge concerning suicide and persons with suicidal thoughts respectively.

After having heard the vignette, participants were asked to what extent they would agree to the following statement: “I would feel and think the same as that person when being in the same situation.” Using a 4-point Likert scale ranging from 1 “strongly disagree” to 4 “strongly agree”, a continuum of self-distinction could be assessed.

Concerning mental health literacy, questions on signs of suicidal thoughts, causes of suicidality, offers of care and treatment options (availability and effectiveness) were asked.

Further, we used the short form of the Literacy of Suicide Scale (LOSS-SF) [42]. Items of the LOSS-SF consist of true and false statements about suicide and suicidal thoughts. Participants state whether they believe these statements are true or false.

In terms of measuring participants’ attitudes towards persons with suicidal thoughts, several instruments that measure stigma of mental illness were used:

The Desire for Social Distance Scale [43] assesses a person’s disposition or reluctance to socially engage with a certain group of persons. The scale contains seven items, each representing a social relationship: tenant, co-worker, neighbour, person one would recommend for a job, person of the same social circle, in-law, and child-carer. Respondents indicated on a 4-point Likert scale to what extent they would accept a person with suicidal thoughts ranging from 1 “strongly disagree” to 4 “strongly agree”.

Further, respondents were asked about their emotional reactions towards affected persons using a list of nine items representing several ways of responding to a person with suicidal thoughts. On a 4-point Likert scale coded from 1 (“strongly disagree”) to 4 (“strongly agree”), respondents stated their agreement to the statements covering the dimensions ‘anger’ (e.g. “I react angrily”), ‘fear’ (e.g. “He/she scares me”) and ‘pro-social’ reactions (e.g. “I feel sympathy”), which were yielded in former principal component analyses [44, 45].

Additionally, we used the short form of the Stigma of Suicide Scale (SOSS-SF) [46]. The SOSS-SF comprises 16 descriptors of a “typical” person who dies by suicide, covering three factors: ‘stigma’, ‘isolation/depression’, and ‘glorification/normalization’. Participants state on a 5-point Likert scale (strongly disagree, disagree, neutral, agree, strongly agree) to what extent they agree with the attributing descriptor (e.g. ‘brave’, ‘isolated’, ‘stupid’). Since the whole questionnaire in this study focused on persons with suicidal thoughts, we modified the original wording from “people who commit suicide” to “people who have suicidal thoughts”.

Additionally, we collected the socio-demographic variables age, gender, education and occupational status as well as religious denomination.

### Statistical analysis

Group differences on suicide stigma between the different vignettes are assessed. Normal distribution is tested using the Kolmogorov-Smirnov-test. Group differences in means are tested for non-parametric and categorical data using the Mann-Whitney-U-test and Chi^2^-test respectively. For parametric data, the t-test is used to compare two groups respectively to conduct an analysis of variance (ANOVA) to draw comparisons between more than two groups. Correlations between more than two variables are examined with multiple linear regression analyses. The unstandardized B-coefficient, the Beta-coefficient, significance and the explained variance (R^2^) are considered.

All analyses are conducted with the statistics software IBM SPSS 25 [47]. For all analyses, results with *p* ≤ 0.05 are considered statistically significant.

## Aim 2: development of e-mental health interventions

### Involvement of affected persons and their relatives

Persons affected by suicidality and relatives of suicidal persons are involved during the whole developmental process of the e-mental health interventions. In German-speaking countries the term “trialogic” or “trialogue” describes the exchange between persons affected by a health problem, relatives/close persons (e.g. friends, family members), and professionals [48–50]. Recruitment was done via the trialogic assembly *“Irre menschlich Hamburg e.V.”* [51, 52]. This lived experience team reviews all text materials in a structured process before its online release. In the interactive online intervention, the lived experience team contributes digital postcard messages, written experience reports, and videos in which they share their personal experience regarding suicide (personal stories).

Members of the lived experience team are at least 18 years old, are given a Participant Information Form, and provide informed consent. Members decide the extent of their participation and have the right to revoke their participation in the project at any time (including the provision of the video and text material).

### Development of evidence-based health information

The method of developing evidence-based health information conforms to international and national [53] quality criteria for the creation of online health information and decision guidance. A methodological paper is developed on this basis and comprises the following aspects [54]:

- Sources are national [55] and international [56] guidelines and systematic reviews.
- During the development and evaluation of the material, persons affected by suicidality participate through the collaborative involvement of self-help organisations, trialogic organisations or patient associations.
- Fact sheets include the development date and the date for the next revision. All information is reviewed at least once a year and revised if required.
- All persons involved in the development of health information are advised to represent only the interests of their delegating organization.
- Experts in the specific area are involved in content development. The authors of a text and their qualifications are named. Experts, and members of the lived experience team assess the text material in a structured peer-review process.

In developing the e-mental health interventions we also consider media guidelines for suicide reporting [57]. Evidence-based health information on suicidality is used for the extension of https://www.psychenet.de/ as well as for the interactive online intervention.

### Development of the interactive online intervention

The interactive online intervention has been developed on the basis of the design, lessons and evidence from the existing Australian project *The Ripple Effect* [31, 40]—an intervention with a focus on rural farming populations. The current intervention content has been translated and adapted to the German cultural context and the focus has been broadened to a general population sample [31].

The project team of *The Ripple Effect* has provided advice on the development of the current intervention. A close collaboration (skype conferences, multi-day face-to-face workshop) with the Australian team has been conducted to build on their groundwork and experience when developing and evaluating the interactive online intervention.

A web design agency is responsible for technical implementation and the design of the intervention. Responsive design, which makes the intervention render well on a variety of devices, like smartphones or tablets, will be applied to enhance user-friendliness.

### Content of the interactive online intervention

The interactive online intervention consists of five chapters (as described in Table 1). A combination of core content and optional content allows participants to choose the level of detailed information preferred.

**Table 1:** Content of the interactive online intervention

Chapter 1: Psychoeducation	
- Evidence-based health information: meaning of suicide respectively suicidality, frequency of suicide, possible causes of suicidality, warning signs, precipitating events, risk- and protective factors	
- Werther- and Papageno effect	
- Suicidality as a continuum	
- Suicide taboo: meaning and function of a taboo in general and for suicide in particular, reasons for tabooing suicide	
- Suicide stigma: meaning of stigma and stigmatization in general and concerning persons with experience of suicide, self-stigma, suicidality as consequence of stigmatization, difference between experienced and anticipated stigmatization, suicidality in various situations (migration background, serious physical dieseases, higher age, homosexual or bisexual orientation, transgender)	
- Selected results of the representative population survey	
- Falsities concerning suicidality opposed to reality	
Chapter 2: Experience reports on suicidality	
- Video reports and text messages by persons with an experience of suicide: e.g. understanding suicide attempts or thoughts, helpful strategies to deal with suicidality from the perspective of affected persons (e.g. “What helped me to deal with suicidal thoughts?”; “What helped me to deal with the suicide of a close person?”)	
Chapter 3: Strategies I - Behavior, Body, Mind, Feelings	
Strategies to deal with suicidality or stigmatization:	
- Explaination of the link between behavior, body, thoughts and feelings	
- Behavior: link between activity and well-being, creating a personal list of positive activities	
- Body: Progressive muscle relaxation	
- Mind: cognitive restructuring technique, questioning stigma related thoughts	
- Feelings: Psychoeducation about feelings, feelings connected with stigma, Mindfulness technique	
Chapter 4: Strategies II - Communication	
- How to talk about suicidality: communication tips	
Chapter 5: Personal goal setting	
- Personal goal setting according to “SMART” criteria (specific, measurable, agreed, realistic, and time specific). Three personal goals can be set.	

Content of the interactive online intervention is tailored for all five chapters depending on the participant’s experience of suicide: suicide attempt in the past, having suicidal thoughts, having lost a close person by suicide, fearing the loss of a close person by suicide, or interested in the topic in general. For example, a participant of the online intervention who lost someone by suicide will be presented with different communication tips (chapter 4) than a participant who fears losing someone by suicide.

Referral to external support services will be provided via online links and telephone numbers of national and regional services, crisis lines and locations of emergency mental health services. Information on support services will be available from every page of the online intervention.

From chapter 2, participants in the interactive online intervention will be able to read and/or write digital postcard messages about individual experiences of suicide and leave a message to other participants. The digital postcard messages will be screened before being included in the online intervention to ensure they comply with media guidelines for talking about suicide [57].

Participants can successively work on five chapters of the interactive online intervention and divide their time as prefered. Overall, an estimated time of 1.5–3 h will be required to complete the intervention. Participants can pause at any time and continue working at the point where they left off. The period over which participants can work on the intervention is flexible, with an approximate guideline of two to four weeks being recommended.

## Aim 3: evaluation of the e-mental health interventions

### Web analytics

Web analytics tool Matomo (https://matomo.org/) will be used to record data including number of visitors, page views, average time on website, bounce rates or access paths. These will be collected for all elements of the e-mental health interventions (self-test, interactive online intervention, information about support services).

**Fig. 1:**
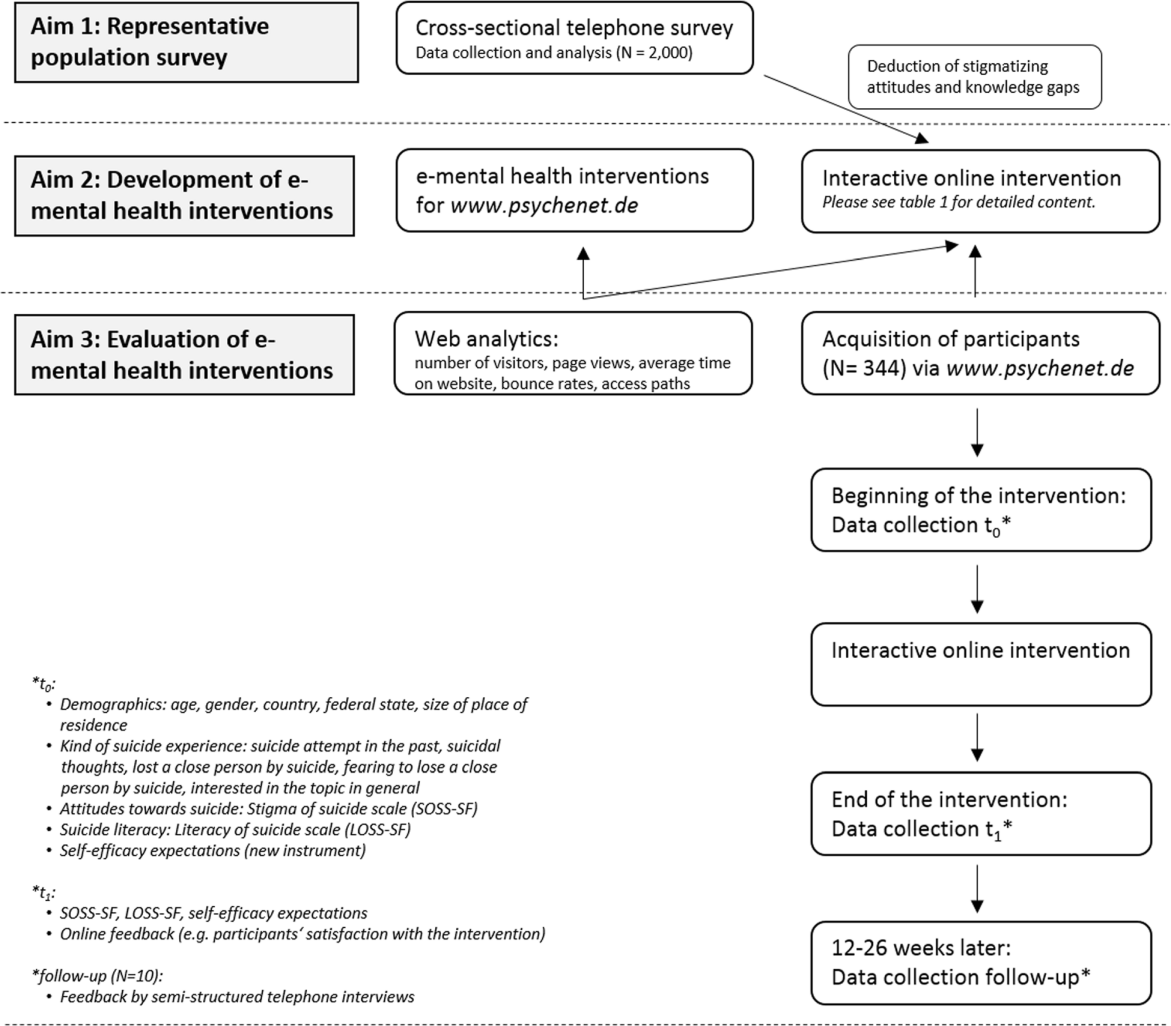
Overview of the project process

Further evaluation steps refer solely to the interactive online intervention. For an overview of the entire project please see Fig. 1.

### Study design

The evaluation of the interactive online intervention draws on the evaluation, lessons and evidence of *The Ripple Effect* [31]. A mixed methods design with three measurement points will be realized. Prior to commencing the interactive online intervention (t_0_), sociodemographic and clinical data, attitudes and knowledge on suicidality (SOSS-SF and LOSS-SF) are collected. An interval-scaled questionnaire will also be developed—based on Bandura’s self-efficacy concept [58, 59]—to assess self-efficacy expectations when dealing with psychologically difficult situations. Psychometric properties of the new survey instrument will be described using the intervention sample. On completion of the intervention (t_1_), a post-survey will be conducted using SOSS-SF and LOSS-SF again (interval between t_0_ and t_1_ is dependent on the time participants take to complete the intervention). At a third time point (t_2_) (12–26 weeks after completion of the intervention), follow-up telephone interviews will be conducted with ten participants who agree to participate in a semi-structured telephone interview.

Due to the exploratory nature of the survey (first use of the intervention and survey tools in the German-speaking region) and the aim to allow participation from all interested persons, we decided against a randomised controlled design. The influence of the intervention on stigma and knowledge is examined, with a pre-post-design, according to the Australian example [31]. Further, qualitative information (e.g. individual experiences with the intervention) will be gathered in free-text fields as well as in follow-up interviews.

### Study sample: inclusion and exclusion criteria

Target group are adults (18 years or older) who have been affected by suicidality themselves or as close persons of those affected. Other interested people can participate as well. The nature of suicide experience is asked at the beginning of the intervention and is considered in the analysis.

In order to participate in the intervention, participants need internet access. Due to the fact that the materials are written in German, people who do not speak or understand the German language will be excluded.

### Acquisition of participants

Participants will be recruited via the e-mental health portal *psychenet.de* [36]. The portal has approximately 80,000 unique visitors per month. In a previous online survey, more than two thirds of users evaluated the *psychenet* website as good or very good [38]. Persons identified as having suicidal thoughts via the PHQ-9 [39] will receive information about their personal risk, support services and about the interactive online intervention (provided free of charge on a subdomain of *psychenet.de*)*.* The interactive online intervention and linked study will be promoted across several areas of the portal (e.g. homepage, disease-specific fact sheets, help section).

### Sample size calculation

As for *The Ripple Effect* [31], a conservative power calculation was performed (not accounting for repeated measures). In a pre-post comparison a sample size of *N* = 241 will be necessary to identify an effect size of d = 0.20 with a power of 0.80 and a significance level of α = 0.05 (dependent t-test, alpha adjusted for two endpoints: SOSS-SF and LOSS-SF). Assuming a dropout rate of 30%, a sample of *N* = 344 is needed at the beginning of the intervention (t_0_).

### Outcome measures

#### Primary outcome measures

Primary outcome measures will be change in suicide stigma and suicide literacy, measured quantitatively using the validated assessment tools SOSS-SF [46] and LOSS-SF [42] (pre- and post-completion of the online intervention).

Similar to *The Ripple Effect* [31], we will adapt the SOSS-SF from a general measure of suicide stigma to assess (1) negative attitudes towards oneself because of own suicidality (self-stigma), and (2) thoughts about how others think of suicidal persons (perceived-stigma).

#### Secondary outcome measures

A secondary outcome measure will be self-efficacy expectations of dealing with psychologically difficult situations. This will be measured quantitatively using an interval-scaled instrument newly developed for this study (pre- and post-completion of the online intervention).

Satisfaction with the intervention will be surveyed quantitatively using Likert-scales, and qualitatively using free-text responses immediately after completion of the intervention (t_1_) and semi-structured follow-up interviews. Follow-up interviews will also explore helpful strategies to reduce stigma and improve knowledge.

### Statistical analysis

For pre-post comparisons (SOSS-SF, LOSS-SF, and self-efficacy expectation scores), we will use the t-test for dependent samples (all three scales are interval-scaled). Kolmogorov-Smirnov test will be used to test normal distribution. Analyses will be conducted for participants who have completed pre and post measurement of SOSS-SF, LOSS-SF, and self-efficacy expectation (“completers”). In case of single missing values, the restricted maximum likelihood-method will be applied. Age, gender and nature of suicide experience (self-afflicted, close person like a friend or family member, interested person) will be considered for subgroup and regression analyses. Participants who have started the intervention but have not completed will be compared to completers in terms of age, gender, and nature of suicide experience, provided data is available. To access the acceptance of the intervention, dropout rate will be calculated. Satisfaction with the intervention will be evaluated descriptively (mean values and standard deviations). All analyses will be conducted using the statistics program IBM SPSS 25 [47]. For all analyses α ≤ 0.05 will be considered statistically significant.

## Discussion

This project aims to develop and evaluate e-mental health interventions to improve knowledge and reduce self-stigma (internalized negative emotional reactions or stereotypes) and perceived-stigma (expected reaction of the public to one’s experience) of persons with an experience of suicide (being affected by suicidality themselves or as close persons of those affected). Persons with a general interest in suicide will be included to broaden the preventative approach.

The nationwide telephone survey helps to identify and understand suicide stigma in the German population. In addition, the survey detects knowledge gaps about suicide which will be addressed by the e-mental health interventions. Thus, this intervention will contribute to an increase in mental health literacy, and suicide literacy in particular which can motivate affected persons to seek support. Further, the online intervention aims to reduce self- and perceived-stigma of affected persons. To ensure the continuation of the intervention, the online intervention will remain on the platform *psychenet.de* after the end of the study (providing the intervention demonstrates achievement of its aims).

### Strengths and limitations

#### Nationwide telephone survey

Telephone surveying has the benefit of accessing a large sample in an efficient manner. Further, the use of the Kish selection grid (to select a random person in households with several residents) and the computer-generated telephone numbers ensure that the sample is drawn from all persons with a telephone. The large number of participants and the representativeness of the sample allow a reliable estimation of the current knowledge and attitudes concerning suicidality in the German population. Thus, content and material of the online intervention can be adapted precisely.

The SOSS-SF and the LOSS-SF are tools to measure suicide stigma and suicide literacy. Since there are no validated instruments measuring suicide stigma and suicide literacy in German, they were translated, culturally adapted, and applied for the first time in the European region. Further, the instruments were used for the first time with a representative sample and via telephone.

As the survey is conducted in Germany, conclusions can only be drawn for the German speaking residential population and data cannot be generalized to other countries. Because suicidality is a very sensitive and taboo topic and telephone interviews may be considered impersonal, socially desirable answers are possible. Further, we cannot rule out a selection bias due to the exclusion of persons with neither a landline nor a cell phone—although the proportion of households with telephone in Germany is high (90.9% landline, 95,5% cell phone [60]).

#### E-mental health interventions

All German-speaking adults with internet access can participate in the online intervention. The material provided in this online intervention is developed trialogically [50, 51], following a structured process with high quality standards. Thus, high quality and evidence-based content will be provided. This addresses a major weakness of some existing suicide preventions sites: A Canadian study found that over half of the statements on such websites were not evidence-based [61]. A more recent study evaluating search engine results when searching for help in a suicidal crisis [62] found that irrelevant websites are identified as well as websites expressing mixed or neutral attitudes towards suicide, or even pages which can be considered as harmful, e.g. describing lethal methods [63].

The online information and the online intervention aim to reach as many participants as possible without exclusion. Therefore no specifc target-group is defined, which is different to the Australian project which focused on male farmers aged 30–64 but did not exclude anyone over 18 years. However, material will be tailored to participant’s experience of suicide: persons who attempted suicide, persons having suicidal thoughts, persons who fear losing someone by suicide, and persons who have lost someone by suicide. Further, evidence-based information on factors that influence suicide risk—such as migration background, serious physical diseases, or sexual orientation—will be provided.

Nevertheless, when a specific population is addressed, life situations of the target group can be taken into account more precisely. Thus, material is adapted for example in terms of language or images (e.g. special design characteristics for young people) and the target group can be contacted in their environment. In the Australian project for example, information on the project was provided via farmer associations and images depicted the type of farming the participants identified with [31].

Selecting a survey tool was difficult, given limited availability of well-evaluated suicide stigma scales measuring self-stigma and perceived-stigma. We wonder if stigma scales may have the effect of reproducing or reinforcing stigmatizing attitudes. While stereotypes, prejudices, and discrimination already exist in society, will answering suicide stigma scale items exascerbate negative beliefs about the self, the world, or the future? Will participants react to stereotypes presented in the phone survey? Moreover, will participants who previously experienced minimal stigma experience an increased belief that people may devalue them because of a mental health crisis?

Our decision to use the SOSS-SF [46] has been based on the ability to compare results with the Australian project *The Ripple Effect* [31]*—*research that has informed the development of our online intervention. We will add a new instrument to measure self-efficacy expectations of dealing with psychologically difficult situations in order to explore the online intervention’s potential to empower users. Whether a short online intervention can change self-efficacy expectations, which may interrelate with the stable trait of participant’s general self-efficacy expectations, remains to be seen.

Although the online intervention is not a substitute for a professional mental health consultation, it can reach persons with limited access to health care (e.g. in rural areas). Furthermore, people who refuse to seek out traditional services, especially those who fear being hospitalized or taking medication, may utilize technology-based mental health services [64]. Thus, the online intervention serves as an opportunity to inform participants about suicidality and to improve health behaviours with reduced barriers.

Presumably, the intervention will most likely reach people who are seeking information about suicidality on the internet. This self-selection is likely to exclude people who are not looking for this information, which may be confounded by particular characteristics of the groups. Although the provided materials will have an engaging and interactive design (e.g. through the use of videos, digital postcard messages, and simple phrasing), the intervention has a quite academic nature. The intervention may be used by persons who have been mentally strained for a long time with extensive internet research experience. These persons may already have high mental health literacy and further improvement, through intervention participation, may lead to a ceiling effect.

The intervention targets participants who, on the one hand, want to deal with suicidality but, on the other hand, are currently not suicidal. In an acute crisis, the intervention does not provide crisis support and may be inappropriate (which is clearly emphasized during the intervention).

The required login to the intervention has advantages and disadvantages. On the one hand, it offers protection of the material as well as assistance with managing the data. On the other hand, the login might also be a barrier to participation.

Due to the exploratory design of the study and the goal to provide an intervention that is accessible and available for all interested parties, a randomized controlled design will not be conducted. Given this, changes in knowledge and stigma will not be causally attributable to the intervention. To test whether this intervention is more or less helpful than no or another intervention, randomized controlled trials are recommended for future research. Future research may also consider revising the intervention content after accounting for the results of this study. After revision, persons interested in the intervention (e.g. a target group of interest identified by this study) could be randomly assigned either to a waitlist control group or an intervention group.

Development of the online material has been conducted in a trialogical exchange process of experience. The collaborative involvement of persons with an experience of suicide in videos and written messages provides credible and relevant content, e.g. the personal reports show that other persons can be in a similar life situation and how they have dealt with their situation. In order to reduce stigma, to increase awareness, and lift the taboo on suicidality, it is important that various parties shed light on the complexity of the phenomenon of suicide. An intervention based on the guiding principle of trialogue presents the perspective of persons affected by suicide as equal to expert opinions and thus emphasizes knowledge and abilities as well as autonomy and maturity of people seeking help. This can be considered as a strength of the project.

The online intervention targets cognitive, emotional, and behavioral components: psychoeducative text material addresses the participants on a cognitive level, whereas personal video stories and written messages about lived experiences of suicide can address participants emotionally. Finally, the personal goal setting and the possibility to leave own digital post cards can stimulate participants into taking action. A recent review includes fourteen e-mental health studies aiming to reduce symptoms associated with suicidality (e.g. suicide ideation, self harm). The online interventions were associated with reductions in suicide ideation at post-intervention. However, only five studies included in the review were developed specifically for self-management of suicidal ideation; the majority of the programs was developed for self-management of depression [65]. Besides *The Ripple Effect* to our knowledge, no other e-mental health approaches to reducing suicide stigma have been conducted to date. Thus, this project will provide important information about the effectiveness of online interventions aiming to reduce suicide stigma and increase suicide literacy.

## Abbreviations

ANOVA: Analysis of variance
CATI: Computer assisted telephone interview
DGPPN: Deutsche gesellschaft für psychiatrie, psychotherapie und nervenheilkunde
LOSS-SF: Literacy of suicide scale - short form
PHQ-9: Patient health questionnaire (depression module, 9 items)
SOSS-SF: Stigma of suicide scale - short form
